# Combined Avulsion Fracture of the Tibial Tuberosity and Lateral Tibial Plateau in an Adolescent: Case Report

**DOI:** 10.5704/MOJ.1303.006

**Published:** 2013-03

**Authors:** S Javed, B Barkatali, M Siddiqui, R Sarin

**Affiliations:** Department of Trauma and Orthopaedics, Royal Blackburn Hospital, Blackburn, United Kingdom; Department of Trauma and Orthopaedics, Royal Blackburn Hospital, Blackburn, United Kingdom; Department of Trauma and Orthopaedics, Royal Blackburn Hospital, Blackburn, United Kingdom; Department of Trauma and Orthopaedics, Royal Blackburn Hospital, Blackburn, United Kingdom

## Abstract

Avulsion of the tibial tuberosity is uncommon. It is usually an athletic injury, accounting for less than 3% of all epiphyseal injuries. We report the case of an avulsion fracture of the tibial tuberosity with unusual articular involvement of the lateral tibial plateau treated with open reduction and internal fixation using cancellous screws. The result was excellent, with complete union of the fracture site, full range of movement at three months and return to normal athletic activity within six months with no complications.

**Key Words:**

tibial tuberosity; avulsion fracture; tibial plateau; adolescent

## Introduction

Avulsion fracture of the tibial tuberosity is a relatively uncommon injury, with reported incidence ranging from 0.4% to 2.7%^1^. Such avulsion occurs when patellar ligament traction exceeds the combined strength of cohesive forces within the apophyseal cartilage, the surrounding perichondrium and the adjacent periosteum 2. Avulsion typically involves one of two mechanisms of injury: violent contraction of the quadriceps muscle against a fixed tibia as occurs in jumping, or acute passive flexion of the knee against the contracted quadriceps^1^. Associated injuries may involve the surrounding ligaments, menisci and rarely a tibial plateau fracture as reported in our case^3^.

## Case Report

A 16-year old male sustained an injury to his left knee during a tackle whilst playing football. Although, he was unable to recall the precise details of the mechanism of injury, he thought his knee was straight at the time of the tackle and his lower leg was forced externally. At presentation, the patient was unable to weight bear and physical examination revealed a closed injury with overlying skin contusions laterally. He had a large knee haemarthrosis and was tender over the tibial tuberosity and lateral joint line and was unable to perform a straight leg raise. Radiographs revealed a Watson-Jones type III avulsion fracture of the tibial tuberosity apophysis and an Aitken type II fracture of the lateral tibial plateau with slight malrotation (Figure 1a & 1b and Table I). Radiographs were reviewed by two orthopaedic consultants and in view of their good quality and subsequent intraoperative knee arthroscopy, a computed tomography (CT) scan was not requested.

We initially performed arthroscopy to assess the joint surface, menisci and cruciate ligaments; this was followed by open reduction and internal fixation via a midline incision, and lateral parapatellar approach. The menisci and ligaments were all intact following arthroscopic examination. Intra-operatively, we found a tibial tuberosity apophysis fracture with a large anterolateral tibial plateau fragment, a single split and no comminution. We saw no concomitant avulsion of the patella tendon from the fragment, though there was significant disruption of the retinaculum laterally. Definitive fixation was achieved with five 4.5mm partially threaded cancellous screws placed under fluoroscopic guidance (3 screws for the tibial tuberosity and 2 for the lateral tibial plateau) with care taken to ensure that the screws did not touch the physis.

Postoperatively, the patient was immobilised in a cylinder plaster cast and allowed toe-touch weight bearing for six weeks. Thereafter, a hinged knee brace was applied for the following six weeks to allow weight bearing, controlled knee flexion and physiotherapy. The patient regained full range of motion by twelve weeks and went on to make an uneventful recovery with a return to normal athletic activity at six months with complete union of the fracture and no growth plate damage or mal-alignment based on serial radiographs and clinical examination over the next 24 months (Figure 2a & 2b).

## Discussion

The tibial tuberosity develops from a secondary ossification centre in the proximal tibia between 7 and 9 years of age. The proximal tibial epiphysis develops in compression and the tibial tuberosity, an apophysis, develops in traction^4^. During ossification, columnated cartilaginous cells with poor tensile strength transiently replace the fibrocartilage, predisposing the tibial tuberosity to traction injury just before or during the later stages of physiologic epiphysiodesis^1^.

The aim of treatment for avulsion fractures of the tibial tuberosity is to restore the extensor mechanism and the joint surface^1^. This is even more important if there is an associated tibial plateau fracture. Improper surgical technique may predispose to fracture related complications such as genu recurvatum and/or leg length discrepancy as well as early degenerative changes. Genu recurvatum is a rare complication of this injury that occurs in patients close to skeletal maturity; in fact, only one case has been reported in the literature^2^.

Undisplaced fractures may be treated with cylinder cast immobilisation. Minimally displaced avulsions can also be treated conservatively with closed reduction in some cases^2^. Displaced fractures are best managed with open reduction and internal fixation to restore the anatomy and the quadriceps-patella mechanism. It has been suggested that open reduction provokes cartilaginous fusion of the tibial apophysis reducing the risk of recurrence. To accomplish fixation, a midline longitudinal incision is recommended to facilitate possible knee surgery in the future^2^.

In the present case, we used a lateral parapatellar approach via a midline incision to provide adequate exposure of the fracture site and avoid injury to the infrapatella branch of the saphenous nerve. Definitive fixation of tibial tuberosity avulsion fractures can be achieved by transfixing pins or screws, staples, tension bands or even direct suture^2^. We used only partially threaded cancellous screws for repair of the lateral retinaculum. Although internal fixation supplemented with tension band wiring theoretically provides greater fracture stability allowing early joint motion for isolated tibial tuberosity fractures, it is not recommended for use if the patient has associated tibial plateau fractures.

We agree with Ozer and colleagues about the possible mechanism of injury for an associated lateral tibial plateau fracture3. Above, we discussed the mechanism of injury for tibial tuberosity fractures that do not involve the plateau. After evaluation of the lateral anatomical structures and biomechanics of the knee they suggested that external rotation of the tibia relative to the femur along with knee hyperextension is the mechanism such an injury to the tibial plateau. They proposed that as weight is transferred from the lateral meniscus, an avulsion fracture of the lateral tibial plateau rim may occur during forceful hyperextension. Furthermore, the lateral retinaculum may also contribute to this injury through its insertion on the proximal tibial epiphysis by exerting force in extension, thereby explaining the reason for lateral retinaculum disruption in our patient^3^.

In our unit, we use the following protocol for all patients presenting with a tibial tuberosity fracture. We investigate the possibility of an associated tibial plateau fracture or other concomitant injury using plain radiographs and assume low threshold for further imaging or arthroscopy in cases of uncertainty. We would not recommend an open approach without such assessment. For displaced avulsion fractures of the tibial tuberosity associated with a tibial plateau fracture, we recommend open reduction and internal fixation with partially threaded cancellous screws in order to achieve anatomical reduction, rigid fixation and realignment of the extensor mechanism; this is followed by six weeks of cylinder cast immobilisation. Continuous screening with an image intensifier may be required as the leg is rotated in the coronal plane to avoid injury to the physis.

A hinged knee brace should be used for an additional six weeks to facilitate controlled knee flexion, physiotherapy and progressive weight bearing. Progressive rehabilitation of the quadriceps is required after cast immobilisation. Though, early mobilisation attenuates joint stiffness and weakness due to prolonged immobilisation, we do not recommend this before six weeks postoperatively. These patients should be followed up until maturity to ensure that there are no fracture-related complications.

**Table I:**
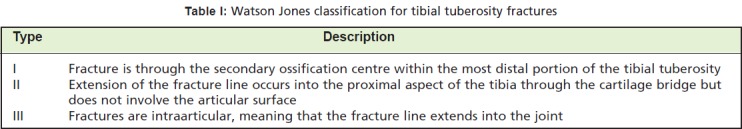
: Watson Jones classification for tibial tuberosity fractures

**Fig. 1a:**
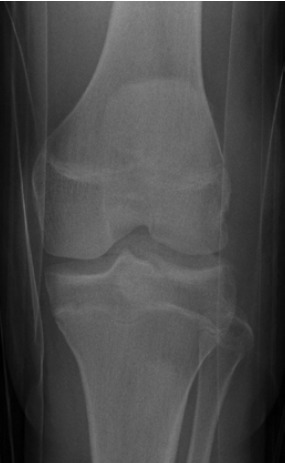
: Preoperative anteroposterior radiograph of the left knee.

**Fig. 1b:**
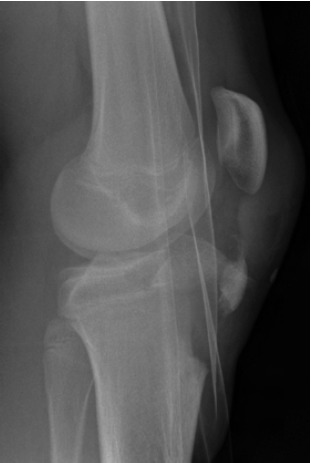
: Preoperative lateral radiograph of the left knee.

**Fig. 2a:**
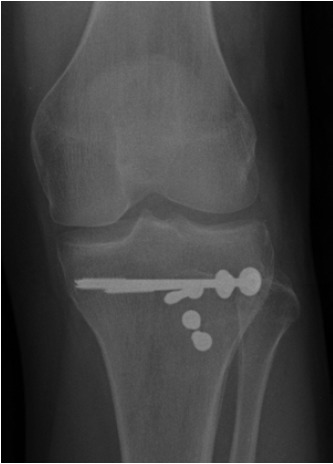
: Post-operative anteroposterior radiograph of the left knee at 24 months.

**Fig. 2b:**
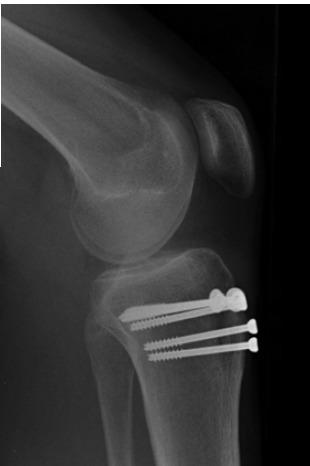
: Postoperative lateral radiograph of the left knee at 24 months.

## References

[1] Frey S, Hosalkar H, Cameron DB, Heath A, David Horn B, Ganley TJ (2008). Tibial tuberosity fractures in adolescents. J Child Orthop.

[2] Nikiforidis PA, Babis GC, Triantafillopoulos IK, Themistocleous GS, Nikolopoulos K (2004). Avulsion fractures of the tibial tuberosity
in adolescent athletes treated by internal fixation and tension band wiring.. Knee Surg Sports Traumatol Arthrosc.

[3] Ozer H, Turanli S, Baltaci G, Tekdemir I (2002). Avulsion of the tibial tuberosity with a lateral plateau rim fracture: case report. Knee Surg Sports Traumatol Arthrosc.

[4] Ogden JA, Southwick WO (1976). Osgood–Schlatter’s disease and tibial tuberosity development. Clin Orthop Relat Res.

